# Transcranial Direct Current Stimulation in the Treatment of Post-Laminectomy Syndrome: A Clinical Trial

**DOI:** 10.1192/bjo.2022.190

**Published:** 2022-06-20

**Authors:** Fernanda Faria, Marília Oliveira, Demosthenes Júnior, Ana Olmos, Camila Cosmo, Gerardo Filho

**Affiliations:** Faculdade de Medicina de São José do Rio Preto (FAMERP), São José do Rio Preto, Brazil

## Abstract

**Aims:**

To evaluate the effectiveness of Transcranial Direct Current Stimulation (tDCS) in treatment of post-laminectomy syndrome.

**Methods:**

Twenty-four patients were randomized in three groups to receive active or sham anodic stimulation (1.5 mA, 20 minutes for five consecutive days, with 25cm2 electrodes) in two different areas (primary motor cortex (M1) vs. dorsolateral prefrontal cortex (DLPFC), according to lateralization of pain. Brief Pain Inventory (BPI) and Visual Analogue Scale (VAS) were instruments used to assess pain, while Clinical Global Impression Scale (CGI) was applied to measure severity disease and clinical response. Additionally, the quality of life assessment was based on World Health Organization Quality-of-Life Scale (WHOQOL-BREF). In order to identify psychiatric comorbidities, Beck's Depression Inventory (BDI) and Beck's Anxiety Inventory (BAI) tests were applied. Comparisons between groups were performed using one-way ANOVA, ANOVA-Welch, Kruskal-Wallis, Man-Whitney, and Fisher's test.

**Results:**

It is observed that there was a statistically significant difference (difference 0,15† [95% CI, 7,07 ± 1,39]) in the way individuals assess their quality of life and the improvement in pain intensity by VAS, especially in M1. The assessment of quality of life among those who showed improvement was higher than those who did not improved.

**Conclusion:**

Application of tDCS in primary motor cortex (M1) produced an improvement in pain pattern in patients with post-laminectomy syndrome. Our data suggest that tDCS - a low-cost, technically simple and highly tolerable technique, is a promising technique for management chronic pain in disorders such as post-laminectomy syndrome.

